# Second Law Analysis for the Experimental Performances of a Cold Heat Exchanger of a Stirling Refrigeration Machine

**DOI:** 10.3390/e22020215

**Published:** 2020-02-14

**Authors:** Steve Djetel-Gothe, François Lanzetta, Sylvie Bégot

**Affiliations:** FEMTO-ST, Energy Department, Univ. Bourgogne Franche-Comté, CNRS Parc technologique, 2 avenue Jean Moulin, 90000 Belfort, France; steve.djetel@femto-st.fr (S.D.-G.); sylvie.begot@univ-fcomte.fr (S.B.)

**Keywords:** Stirling cycle, refrigerator, heat exchanger, second law, entropy production

## Abstract

The second law of thermodynamics is applied to evaluate the influence of entropy generation on the performances of a cold heat exchanger of an experimental Stirling refrigeration machine by means of three factors: the entropy generation rate $N_{S}$, the irreversibility distribution ratio *ϕ* and the Bejan number ${Be|}_{N_{S}}$ based on a dimensionless entropy ratio that we introduced. These factors are investigated as functions of characteristic dimensions of the heat exchanger (hydraulic diameter and length), coolant mass flow and cold gas temperature. We have demonstrated the role of these factors on the thermal and fluid friction irreversibilities. The conclusions are derived from the behavior of the entropy generation factors concerning the heat transfer and fluid friction characteristics of a double-pipe type heat exchanger crossed by a coolant liquid (55/45 by mass ethylene glycol/water mixture) in the temperature range 240 K < *T_C_* < 300 K. The mathematical model of entropy generation includes experimental measurements of pressures, temperatures and coolant mass flow, and the characteristic dimensions of the heat exchanger. A large characteristic length and small hydraulic diameter generate large entropy production, especially at a low mean temperature, because the high value of the coolant liquid viscosity increases the fluid frictions. The model and experiments showed the dominance of heat transfer over viscous friction in the cold heat exchanger and ${Be|}_{N_{S}}\to 1$ and *ϕ* → 0 for mass flow rates $\dot{m}\to 0.1$ kg.s^−1^.

## 1. Introduction

Refrigeration plays a major role in many different sectors, ranging from food, air conditioning, healthcare, industry and, especially energy. Nowadays, the number of refrigeration air-conditioning and heat pump systems in operation worldwide is roughly 5 billion, if we consider 2.6 billion air-conditioning stationary and mobile units and 2 billion domestic refrigerators and freezers [1]. The global electricity demand for refrigeration, including air conditioning, could more than double by 2050 [2]. The Kyoto Protocol was adopted in 1997 and entered into force on 16 February 2005. It aimed to prevent global warming due to the use of HCFC/CFC refrigerants and the objective is to stabilise greenhouse gas concentrations at a level that would prevent dangerous anthropogenic (human-induced) interference with the climate system. It can be seen that developing a cooling system without using HCFC/CFC working fluid is unavoidable in the future. Stirling coolers are used as commercial cryocoolers in the cryogenic field, military applications, liquid air production plants, cooling electronics, carbon capture and domestic applications [3,4,5,6].

Thus, in order to decrease electricity consumption and to prevent global warning, it is necessary to optimize these cooling machines and systems, particularly the heat exchangers. These are systems to transfer heat between two or more fluids at different temperatures and pressures, separated by a heat-transfer surface. The optimal conversion of heat energy involves a heat exchanger in order to minimize the energy consumption and costs regarding several criteria, such as materials, geometries, flow rates, flow arrangements, operating temperatures and pressures, and transient or steady-state operation [7,8,9,10]. In the field of refrigeration, the objective is to extract heat from different products in gas, liquid or solid state with minimum energy cost and maximum efficiency.

**Figure 1:**
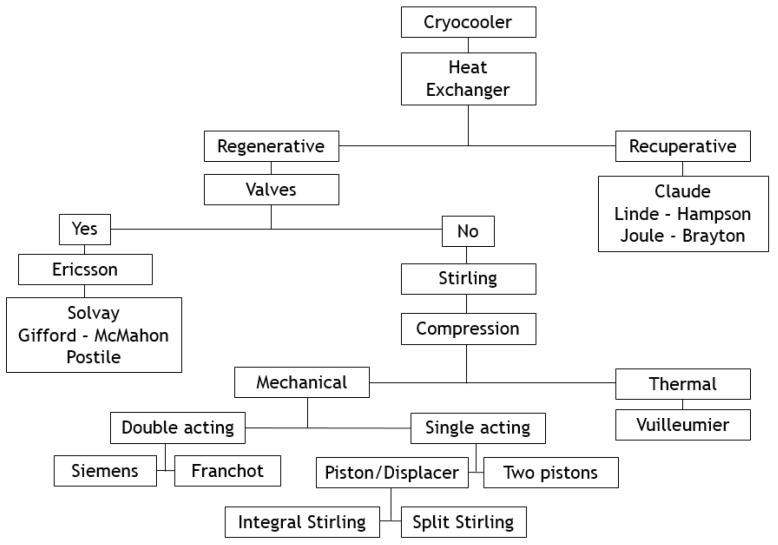
Classification of cryocoolers [12].

**Figure 2:**
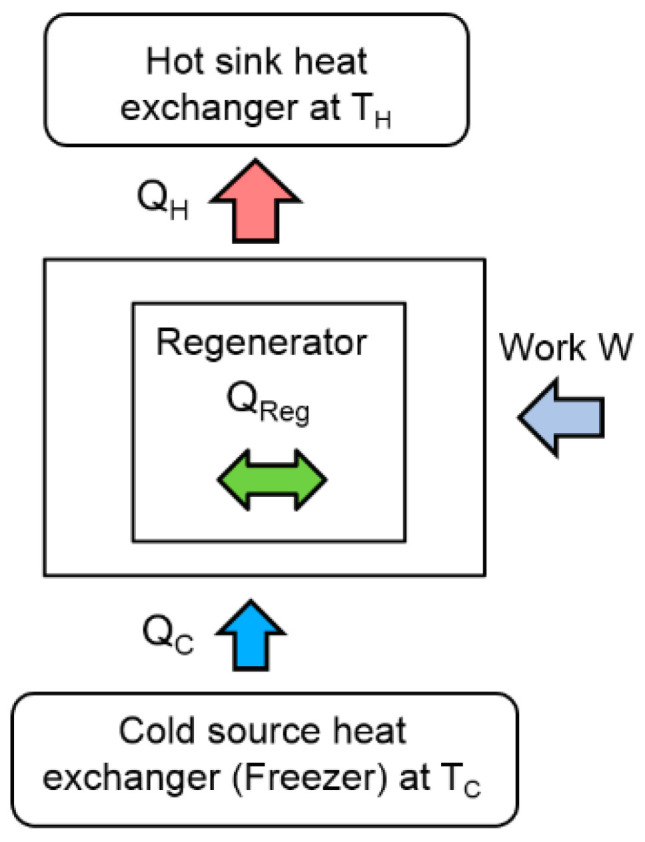
Schematic diagram of an ideal Stirling refrigerator.

**Figure 3:**
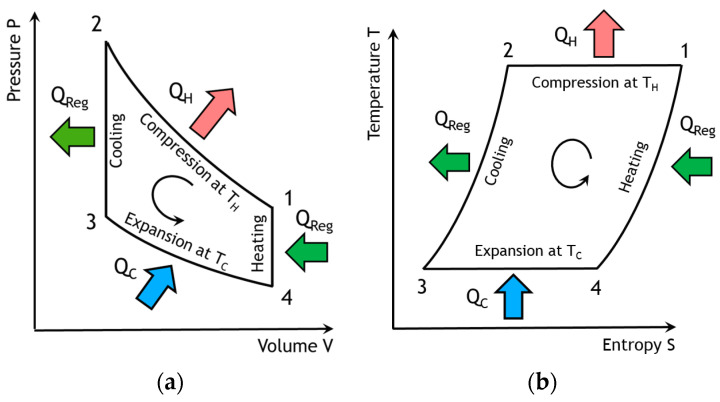
Ideal Stirling refrigeration cycle. (**a**) Pressure-Volume diagram; (**b**) Temperature-Entropy diagram.

**Figure 4:**
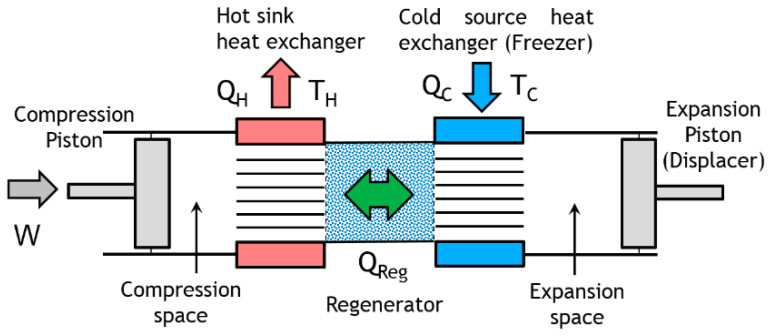
Stirling refrigerator for an alpha architecture.

This paper deals with the optimization of a heat exchanger (the freezer) of a refrigerating machine, used in a Stirling machine working at low and moderate temperatures (between −100 and 0 °C). This machine is a member of the cryocooler’s technology family (Figure 1). Generally, the closed cycle of the Stirling machine concerns engines, coolers and heat pumps [11,12]. The thermodynamic cycle is the same in each case except that the process direction is reversed. In its theoretical refrigerating cycle (Figure 2, Figure 3 and Figure 4), the working fluid is compressed at the highest constant temperature *T_H_* (1–2) and Q_H_ is the corresponding heat rejected to the environment through the cooler exchanger.

The fluid is cooled at constant volume (2–3) by the heat Q_R_ rejected from the gas to the regenerator. Then, the expansion work (3–4) takes place at the lowest constant temperature T_C_ and the external heat Q_C_ is extracted from the surroundings and supplied to the gas through the freezer exchanger. Finally, the fluid is heated from the temperature T_C_ to T_H_ by the corresponding heat Q_R_ stored in the regenerator during the process at constant volume (4–1). Both isochoric processes take place in a porous heat exchanger called the regenerator [4,5], whose efficiency is a key point of Stirling machine performances.

**Figure 5:**
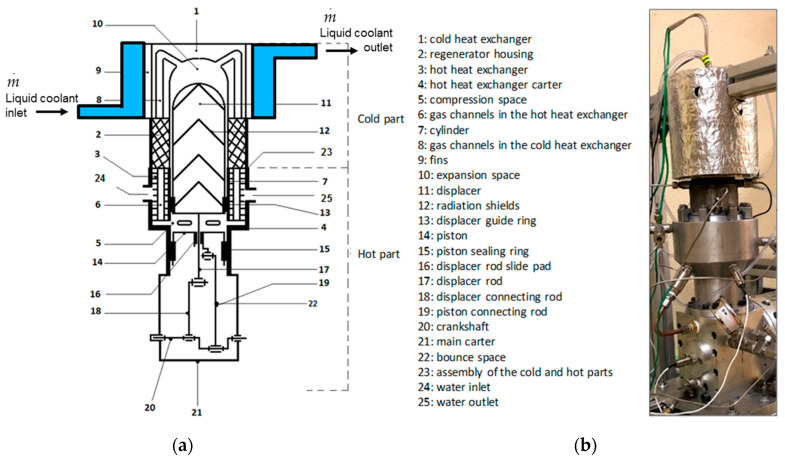
Stirling refrigerating machine [25]. (**a**) Profile of the mechanical configuration; (**b**) The cold heat exchanger is thermally insulated (white material on the photo).

In refrigeration operation, heat is then rejected to the hot sink during the compression (1–2) and provided to the cold source in the compression stage (3–4). In a Stirling machine, these exchangers play a crucial role in the optimization of the performances. The objective of this paper is to optimize the performance of these exchangers by means of the second law of analysis and to study the behavior of the entropy generation. This approach focuses on the cold source exchanger (the freezer) at constant temperature *T_C_* (Figure 5), considering that the same approach could concern the exchanger of the hot sink at temperature *T_H_*.

Entropy generation analysis is considered as a measure of sustainability because a process with a lower entropy generation rate is more sustainable and able to realize energy conversion more efficiently. Nowadays, new indicators based on an engineering approach of irreversibility are used to evaluate both the technological level and the environmental impact of the production process and the socio-economic conditions [13,14,15]. Heat and mass transfer processes and thermal systems like heat exchangers have been studied and optimized in order to minimize the irreversibilities using the second law of thermodynamics in fundamental works published since the 1980s [16,17,18,19]. The second law of thermodynamics method is applied to general convective heat transfer flows in ducts and it defines the entropy rate contributed by heat transfer irreversibilities and fluid flow frictions. Convective heat transfer through ducts is analyzed with constant heat flux or constant temperature [20,21,22,23,24].

The main objective of this work is to determine the optimum duct or heat exchanger geometry which minimize losses for a range of Reynold numbers, hydraulic diameters, and fins geometry (thickness, spacing). We describe the experimental setup based on a Beta Stirling refrigerator, specifically focusing on the cold heat exchanger. Then, we will focus our study on the second law analysis of the experimental performances of the cold heat exchanger. The model and optimization present complementary criteria and alternative ratios, like the dimensionless entropy ratio, irreversibility distribution ratio and Bejan number, that we used in this study to determine the performances of a cold heat exchanger of a Stirling refrigerator.

## 2. Experimental Setup

The Stirling refrigerator in the Beta configuration (Figure 5) is a single-cylinder machine. It consists of two pistons, a working piston and a displacer piston, a compression volume, an expansion volume, a volume occupied by a regenerator, and two heat exchangers. The working piston is used to compress and expand the fluid. The displacer piston function is to displace the gas between the hot and cold parts of the machine. The function of the regenerator is to store the heat of the fluid during one of the isochoric transformations of the cycle and to restore it in the other one. The heat exchanger of the hot part of the machine is water-cooled. The lower part of the machine, called the bounce space, contains the connecting rods and the crankshaft.

The main design parameters of the machine are: mean pressure between 15 and 20 bar, Nitrogen as working gas, stainless steel for the cold head and aluminum for the radiator at the hot sink (Table 1). The experimental cooling capacity is 550 W at 273 K and 280 W at 230 K.

## 3. Mathematical Model

In a heat exchanger, real processes present irreversibilities caused by losses due to fluid friction, heat exchange across a finite temperature difference and heat exchange with the environment [26]. Second law analysis is a method developed to design systems on minimum entropy production caused by these losses [16,27,28]. The second law equation describes the irreversibility of the process, in terms of entropy generation rate ${\dot{S}}_{gen}$ within the heat exchanger between inlet and outlet ducts of a system boundary.

(1) $${\dot{S}}_{gen}=\frac{\partial S}{\partial t}-\frac{\dot{Q}}{T}-\sum_{in}{\dot{m}}_{in}s+\sum_{out}{\dot{m}}_{out}s\geq 0$$

For steady operation (∂S/∂t=0), consider an arbitrary flow passage of length *dx* with a finite temperature difference ∆*T* = (*T_w_* − *T*) between the wall temperature *T_w_* and the bulk temperature *T* of the fluid, the rate of entropy generation per unit length is

(2) $${\dot{S}}_{gen}^{\prime }=\frac{{\dot{Q}}^{\prime }\Delta T}{T^{2}\left(1+\tau \right)}+\frac{\dot{m}}{\rho T}\left(-\frac{dP}{dx}\right)$$

where *τ* = *ΔT*/*T* a dimensionless temperature difference, ${\dot{Q}}^{\prime }$ the wall heat transfer per unit length, $\dot{m}$ the mass flow rate and (−dP/dx) the longitudinal pressure gradient.

The quantity $\frac{{\dot{Q}}^{\prime }\Delta T}{T^{2}\left(1+\tau \right)}$ corresponds to the entropy generation rate accounting for the heat transfer irreversibility (${\dot{S}}_{gen,\Delta T}^{\prime }$) and the term $\frac{\dot{m}}{\rho T}\left(-\frac{dP}{dx}\right)$ corresponds to the entropy generation rate for the fluid friction irreversibility (${\dot{S}}_{gen,\Delta P}^{\prime }$). Based on these quantities, Bejan defined the irreversibility distribution ratio *ϕ* by [29]:

(3) $$\phi =\frac{{\dot{S}}_{gen,\Delta P}^{\prime }}{{\dot{S}}_{gen,\Delta T}^{\prime }}=\frac{\frac{\dot{m}}{\rho T}\left(-\frac{dP}{dx}\right)}{\frac{{\dot{Q}}^{\prime }\Delta T}{T^{2}\left(1+\tau \right)}}$$

The entropy generation irreversibility can be described by the Bejan number *Be* as the ratio between the heat transfer irreversibility and the total irreversibility due to heat transfer and fluid flow [30,31]:

(4) $$Be=\frac{{\dot{S}}_{gen,\Delta T}^{\prime }}{{\dot{S}}_{gen,\Delta T}^{\prime }+{\dot{S}}_{gen,\Delta P}^{\prime }}=\frac{1}{1+\phi }$$

The heat transfer dominates when Be→1 (or ϕ→0) and the fluid friction dominates when *Be* → 0 (or *ϕ* → ∞). The ideal equilibrium between these two irreversibilities is reached for *Be* = 1/2 or (*ϕ* = 1).

Assuming the refrigerating fluid to be incompressible, Equation (2) can be written as:

(5) $${\dot{S}}_{gen}^{\prime }=\underset{{\dot{S}}_{gen,\Delta T}^{\prime }}{\underset{︸}{\dot{m}c_{p}\frac{dT}{dx}\frac{\Delta T}{T^{2}\left(1+\tau \right)}}}+\underset{{\dot{S}}_{gen,\Delta P}^{\prime }}{\underset{︸}{\frac{\dot{m}}{\rho T}\left(-\frac{dP}{dx}\right)}}$$

Equation (6) represents the entropy generation per unit length due to heat transfer across finite temperature difference and to fluid friction, respectively. If we consider that the dimensionless temperature difference *τ* = *ΔT*/*T* ≪ 1, the following equation is obtained:

(6) $${\dot{S}}_{gen}^{\prime }=\dot{m}c_{p}\frac{dT}{dx}\frac{\Delta T}{T^{2}}+\frac{\dot{m}}{\rho T}\left(-\frac{dP}{dx}\right)$$

**Figure 6:**
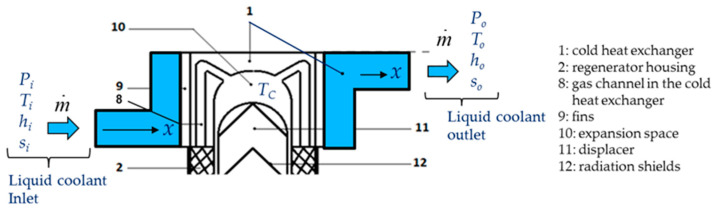
Control volume of the cold heat exchanger.

Derived from Figure 5, the Figure 6 presents the control volume of the Stirling cold heat exchanger for energy and entropy analysis.

The cold heat exchanger is a double-pipe type heat exchanger made up of two concentric circular tubes (Label 1 in Figure 6). The incompressible coolant liquid flows continuously, with a mass flow $\dot{m}$, through the annular passage under the temperature $\left(T_{i}-T_{o}\right)$ and pressure $\left(P_{i}-P_{o}\right)$ gradients, respectively. The other fluid is the working gas (Nitrogen) of the Stirling refrigerator and it flows alternatively (corresponding to the rotational frequency of the machine) at constant temperature *T_C_* in the inner tube corresponding to the cylinder of the machine (Labels 8 and 10). The heat flux $\dot{Q}$ is extracted from the surface and maintained at the constant temperature *T_C_* along the heat exchanger surface. This temperature is measured in the expansion volume of the refrigerator and depends on different parameters like thermal load, pressure of the gas, rotational speed [12,32]. The heat exchanger is considered as a duct at constant temperature *T_C_* with a hydraulic diameter *D_h_* and a total length *L* corresponding to mean distance between the inlet and the outlet of the heat exchanger.

Equation (7) is integrated along the length, and the expression of the entropy generation ${\dot{S}}_{gen}$ becomes [21,22,33]:

(7) $${\dot{S}}_{gen}=\dot{m}c_{p}\left(T_{o}-T_{i}\right)\frac{\left(T_{c}-T_{o}\right)}{T_{i}T_{o}}+\frac{32{\dot{m}}^{3}f}{\rho^{2}\pi^{2}D_{h}^{5}}\frac{L}{T_{C}}$$

where $-dP/dx=2f\rho u_{m}^{2}/D_{h}$ and $u_{m}=4\dot{m}/\left(\rho \pi D_{h}^{2}\right)$.

The hydraulic diameter approach is a simple dimensional parameter method to calculate the heat transfer and the pressure drop in the heat exchanger. The hydraulic diameter *D_h_* of the cold heat exchanger is defined [34].

(8) $$D_{h}=4\left(\frac{\mathrm{minimum}\;\mathrm{flow}\;\mathrm{area}}{\mathrm{frontal}\;\mathrm{area}}\right)\left(\frac{\mathrm{total}\;\mathrm{volume}}{\mathrm{total}\;\mathrm{surface}\;\mathrm{area}}\right)$$

The rate of entropy generation is represented as the dimensionless form *N_S_* [29]:

(9) $$N_{S}=\frac{{\dot{S}}_{gen}}{\dot{m}c_{p}}$$

where $\left(\dot{m}c_{p}\right)$ is the heat capacity rate within the heat exchanger. In this work, the heat capacity rate is the product of the mass flow and specific heat capacity rate of the incompressible fluid.

The fluid flow is considered to be fully developed inside the exchanger. The friction factor *f* is given for a well-known correlation [35,36,37]. The complex flow field generated by the internal geometry of the heat exchanger influences the pressure gradient required to drive the flow. The friction factor *f* is defined

(10) $$f=aRe^{b}$$

with the Reynolds number $Re=\rho u_{m}D_{h}/\mu$. The two constants *a* and *b* depend on the fluid properties, the flow regime and the geometry constraints of the heat exchanger. These two coefficients were identified from data reduction experimental results [38] and pressure drop data were correlated to ±10% for *Re* < 2000 (laminar flow condition).

(11) $$f=34.9\;Re^{-0.775}$$

**Figure 7:**
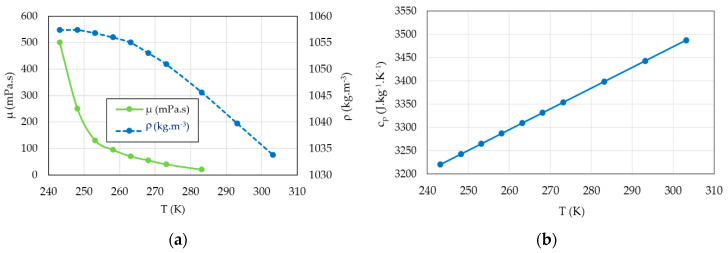
Physical properties of the coolant liquid (55/45 by mass ethylene glycol/water mixture) in the temperature range 240 K < *T* < 300 K [39] (**a**) Density *ρ* (kg.m^−3^) and dynamic viscosity *μ* (mPa.s); (**b**) Specific heat capacity *c_p_* (J.kg^−1^.K^−1^).

The average of the inlet and outlet bulk temperatures is used to calculate the physical parameters (density, viscosity and specific heat capacity) of the coolant liquid (55/45 by mass ethylene glycol/water mixture) in the temperature range 240 K < *T* < 300 K (Table 2 and Figure 7). Ethylene glycol lowers the specific heat capacity of water mixtures relative to pure water.

Considering Equation (10), we get:

(12) $$N_{S}=\underset{N_{S,\Delta T}}{\underset{︸}{\left(T_{0}-T_{i}\right)\frac{\left(T_{C}-T_{0}\right)}{T_{i}T_{0}}}}+\underset{N_{S,\Delta P}}{\underset{︸}{\frac{32{\dot{m}}^{2}f}{c_{p}\rho^{2}\pi^{2}D_{h}^{5}}\frac{L}{T_{C}}}}$$

The first term corresponds to dimensionless rate of entropy production due to the thermal irreversibilities (*N_S,_*_∆*T*_), and the second one, (*N_S,_*_∆*P*_), to the pressure irreversibilities.

From Equation (13), we introduce a Bejan number ${Be|}_{N_{S}}$ based on a dimensionless entropy ratio:

(13) $${Be|}_{N_{S}}=\frac{N_{S,\Delta T}}{N_{S,\Delta T}+N_{S,\Delta P}}=\frac{\left(T_{0}-T_{i}\right)\frac{\left(T_{C}-T_{0}\right)}{T_{i}T_{0}}}{\left(T_{0}-T_{i}\right)\frac{\left(T_{C}-T_{0}\right)}{T_{i}T_{0}}+\frac{32{\dot{m}}^{2}f}{c_{p}\rho^{2}\pi^{2}D_{h}^{5}}\frac{L}{T_{C}}}$$

## 4. Results and Discussion

Both fluid friction and heat transfer contribute to the rate of entropy generation represented by Equations (4), (5), (10) and (13). Entropy generation is investigated with the effects of different parameters concerning the cold heat exchanger (Length *L*, hydraulic diameter *D_h_*, mass flow $\dot{m}$, temperature *T_C_* of the gas inside the cold volume of the Stirling refrigerator). Before examining the dependence of these parameters on entropy generation, it is necessary to discuss the following observations and assumptions. Measurements are performed in the cold heat exchanger (the freezer) of the Stirling refrigerator and concern the temperatures and pressures of the working gas inside the expansion volume corresponding to the internal cold heat exchanger, mass flow and pressures of the coolant liquid (mix water/ethylene–glycol) and rotational speed of the refrigerator (Figure 6).

In this section, we used constant values of inlet and outlet temperatures fixed at *T_i_* = 300 K and *T_o_* = 260 K, respectively. The temperature *T_o_* corresponds to a constraint we need for an industrial process not described in this article. The hydraulic diameter of the cold heat exchanger is *D_h_* = 0.015 m and its characteristic length is *L* = 0.10 m. For the coolant liquid, the variation of viscosity with temperature is responsible for most of the property effects. The coolant fluid flow $\dot{m}$ vary in the range (0.003–0.010 kg.s^−1^). In our study, we performed experiments with two mass flows rates of coolant liquid $\dot{m}$ = 0.003 kg.s^−1^ and $\dot{m}$ = 0.004 kg.s^−1^, respectively. At low temperatures, the viscosity increases significantly and between 243 K and 263 K the dynamic viscosity increases in a ratio of around 7 (Table 2) and has the direct effect of increasing the fluid frictions. We used the film temperature based on the wall and mean bulk temperatures of the liquid flow rate to calculate its properties. We performed the measurements when the machine was operating in steady state at a fixed rotational speed *ω* with 35 rad.s^−1^ < *ω* < 80 rad.s^−1^ and for a given working gas temperature *T_C_* with 190 K < *T_C_* < 273 K. At each extremity of the exchanger (inlet and outlet on Figure 6), we collected the characteristic data of the coolant fluid: the refrigerant mass flow rate $\dot{m}$, the inlet and outlet pressures *P_i_* and *P_o_* and their corresponding temperatures *T_i_* and *T_o_*, respectively. Then, we introduced the experimental data in the thermodynamic model concerning the temperatures, the pressures, the mass flows, the hydraulic diameter, and the mean length *L* of the cold heat exchanger. We finally used the model to extrapolate the performances of the refrigerator in terms of irreversibility distribution ratio *ϕ* (Equation (3)), entropy generation rate *N_S_* (Equations (10) and (13)) and Bejan numbers ${Be|}_{N_{S}}$ (Equations (5) and (14)).

### 4.1. Effect of Characteristic Dimensions (Length L and Hydraulic Diameter D_h_)

**Figure 8:**
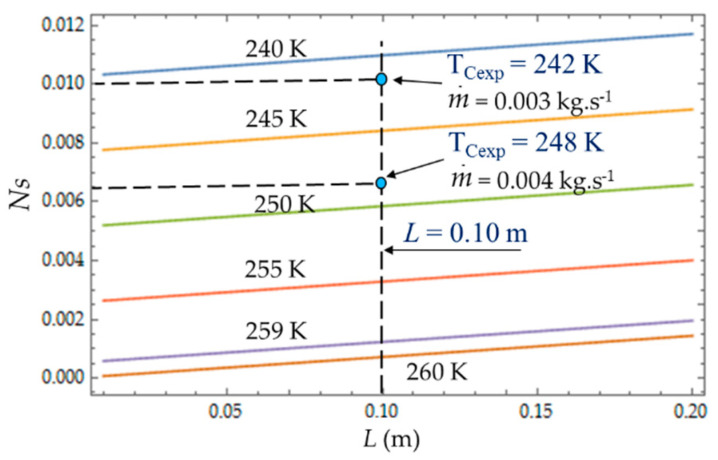
Variations of entropy generation rate *N_S_* as function of cold heat exchange *L* for different temperatures T_C_. Experimental results for *D_h_* = 0.015 m, *L* = 0.010 m, = 0.003 kg.s^−1^ and $\dot{m}$ = 0.004 kg.s^−1^.

**Figure 9:**
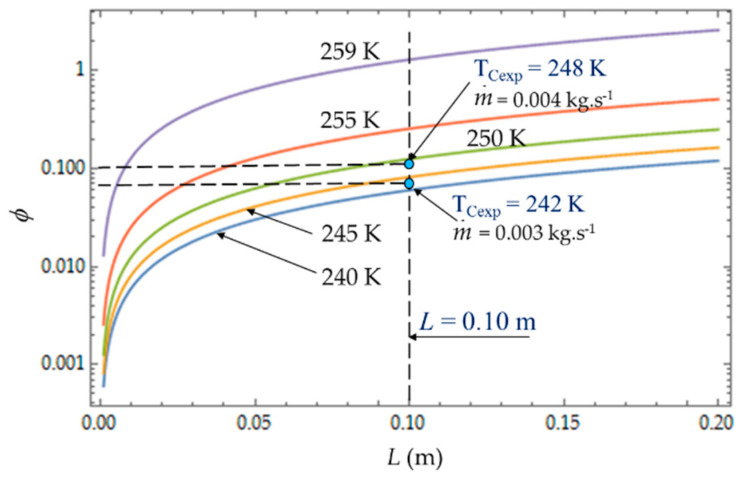
Variation of the irreversibility distribution ratio *φ* as function of cold heat exchange *L* for different temperatures *T_C_*. Experimental results for *D_h_* = 0.015 m, *L* = 0.10 m, $\dot{m}$ = 0.003 kg.s^−1^ and $\dot{m}$ = 0.004 kg.s^−1^.

**Figure 10:**
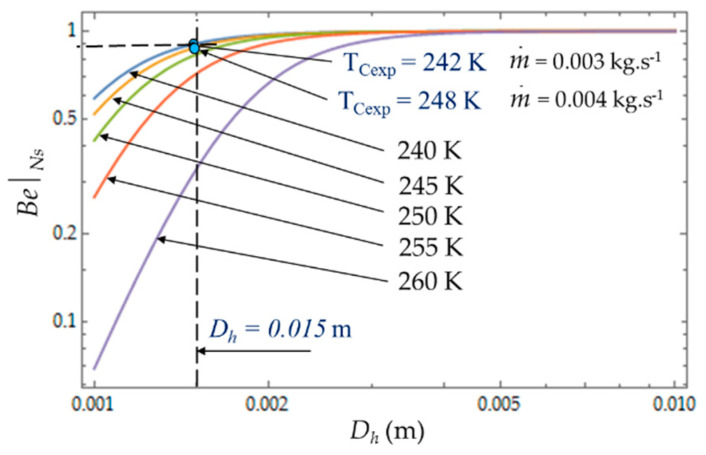
Variations of Bejan number ${Be|}_{N_{S}}$ as function of hydraulic diameter *D_h_* for different temperatures *T_C_*. Experimental results for *D_h_* = 0.015 m, *L* = 0.1 m, $\dot{m}$ = 0.003 kg.s^−1^ and $\dot{m}$ = 0.004 kg.s^−1^.

Figure 8, Figure 9 and Figure 10 present the entropy generation parameters (N_S_, ${Be|}_{N_{S}}$ and φ) as functions of length (L) and hydraulic diameter (D_h_) for fixed values of cold gas temperature (T_C_). The entropy generation number (N_S_) increases with the length L of the heat exchanger because of the increase in the fluid friction and pressure drop. For a given length, N_S_ (Figure 8) increases when the temperature T_C_ decreases because the heat transfer and the entropy production increase. For each mass flow rate $\dot{m}$ of the cooling liquid, we measured the temperature T_C_ of the cold gas inside the expansion space of the refrigerator. For $\dot{m}$ = 0.003 kg.s^−1^, this temperature is *T_Cexp_* = 242 K and it corresponds to the entropy generation number *N_S_* ≈ 0.010, while the theoretical model provides a higher value *N_S_* ≈ 0.011 (+10%) and a lower temperature *T_C_* = 240 K. When the flow rate increases ($\dot{m}$ = 0.004 kg.s^−1^), the pressure drop also logically increases, causing the decrease in $N_{S}$.

The irreversibility distribution ratio φ increases with the length L of the heat exchanger (Figure 9). At given length L, the smallest values of φ correspond to the case for which the heat transfer dominates. We observe at L = 0.10 m (Figure 9), an increase in φ as the mass flow rate $\dot{m}$ increases because the fluid frictions dominate, as was observed in the literature [3,22,40].

**Figure 11:**
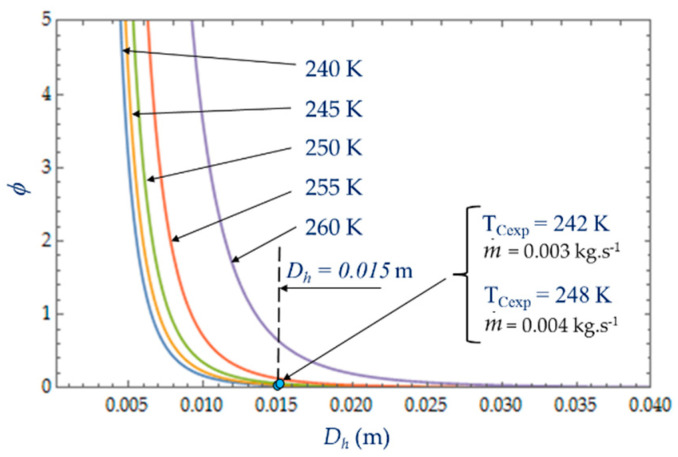
Variation of the irreversibility distribution ratio φ as function of hydraulic diameter *D_h_* for different temperatures T_C_. Experimental results for *D_h_* = 0.015 m, *L* = 0.1 m, $\dot{m}$ = 0.003 kg.s^−1^ and $\dot{m}$ = 0.004 kg.s^−1^.

Figure 10 plots the evolution of ${Be|}_{N_{S}}$ as a function of cold heat exchange length *L* at different temperatures *T_C_*. The limit ${Be|}_{N_{S}}\to 1$ when the heat transfer dominates. At given mass flow rate $\dot{m}$, for small values of hydraulic diameter (*D_h_* < 5 mm), the effect of fluid friction is more effective and ${Be|}_{N_{S}}$ varies with great sensitivity to temperature *T_C_*. For higher values of hydraulic diameter, *D_h_* > 5 mm, the heat transfer dominates fluid frictions regardless of the temperature *T_C_* of the cold volume and, consequently, *ϕ* → 0 (Figure 11). The effects of the variation in the mass flows $\dot{m}=0.003\;\mathrm{kg}.s^{-1}$ and $\dot{m}=0.004\;\mathrm{kg}.s^{-1}$ are of the same amplitude in our experiments and show the dominance of the heat transfer on the fluid frictions (${Be|}_{N_{S}}\to 1$ and *ϕ* → 0).

**Figure 12:**
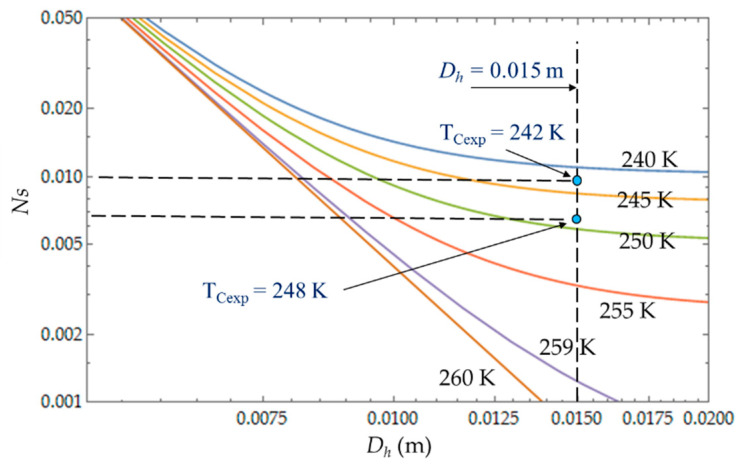
Variations of entropy generation rate *N_S_* as function of hydraulic diameter *D_h_* for different temperatures *T_C_*. Experimental results for *D_h_* = 0.015 m, *L* = 0.1 m, $\dot{m}$ = 0.003 kg.s^−1^ and $\dot{m}$ = 0.004 kg.s^−1^.

The heat transfer impacts the entropy generation rate *N_S_*, which logically decreases with the temperature *T_C_*, and the hydraulic diameter *D_h_*, corresponding to the dominance of the heat transfer irresversibilities over fluid losses (Figure 12). That is what we observed with the two experimental temperatures *T_Cexp_* = 242 K and *T_Cexp_* = 248 K corresponding to the two mass flow rates $\dot{m}=0.003\;\mathrm{kg}.s^{-1}$ and $\dot{m}=0.004\;\mathrm{kg}.s^{-1}$.

The objective is to reduce the degree of irreversibility of the refrigerator and to obtain small values of *N_S_* [16,22,33,41]. This objective depends on the parameters temperature of the cold volume and hydraulic diameter. We observe that the properties of the refrigerant play an important role. When the temperature of the fluid is too low, the viscosity of the fluid and the pressure drops increase and *N_S_* increases as well, which reduces the performance of the refrigerator. For large values of *D_h_*, the entropy generated by the fluid losses is no longer affected by the flow friction losses. Ns tends towards a constant (in our case, this phenomenon is quite obvious for the temperatures 240 K < *Tc* < 250 K) and the irreversibility distribution ratio φ tends to zero (Figure 11). The entropy generation rate *N_S_* depends mainly on the part of the entropy due to heat transfers and then decreases as a function of temperature *T_C_* and hydraulic diameter *D_h_* across a trade-off between fluid flow irreversibilities and heat transfer.

### 4.2. Effect of Coolant Mass Flow $\dot{m}$

**Figure 13:**
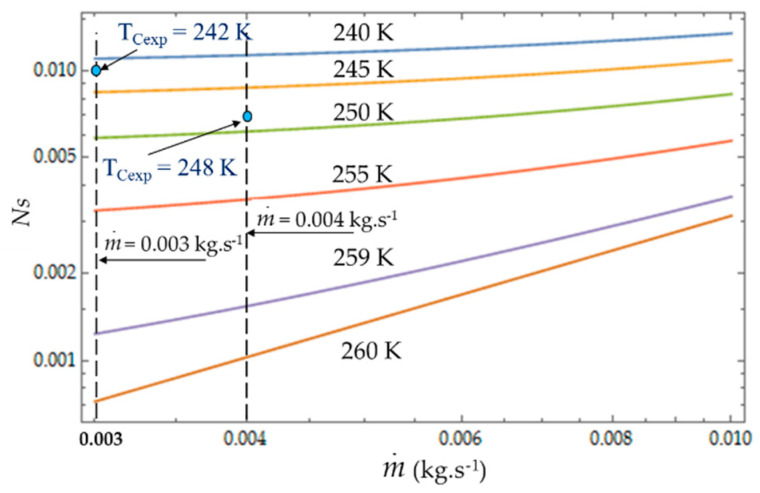
Variations in entropy generation rate *N_S_* as function of mass flow $\dot{m}$ for different temperatures *T_C_* Experimental results for *D_h_* = 0.015 m, *L* = 0.1 m, $\dot{m}$ = 0.003 kg.s^−1^ and $\dot{m}$ = 0.004 kg.s^−1^.

**Figure 14:**
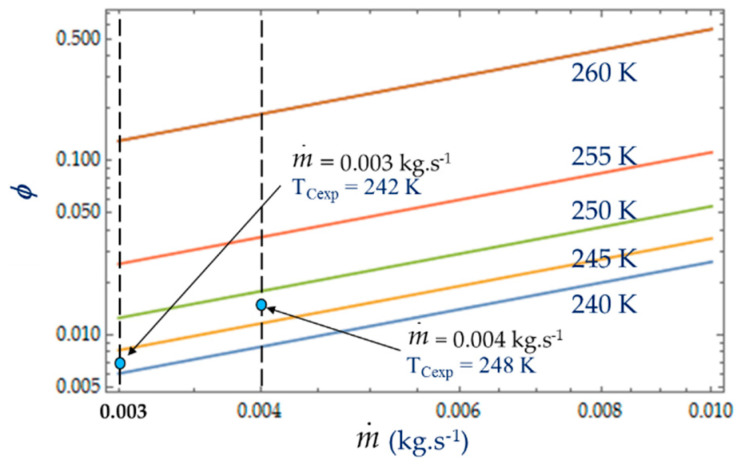
Variation in the irreversibility distribution ratio *φ* as function of mass flow $\dot{m}$ for different temperatures *T_C_.* Experimental results for *D_h_* = 0.015 m, *L* = 0.1 m, $\dot{m}$ = 0.003 kg.s^−1^ and $\dot{m}$ = 0.004 kg.s^−1^.

**Figure 15:**
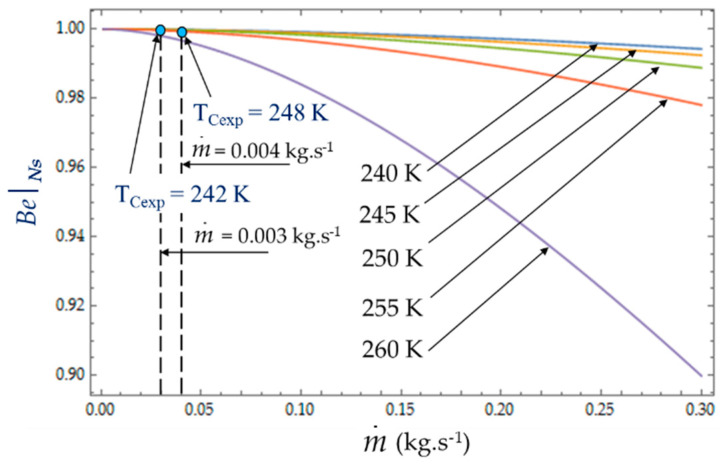
Variation in Bejan number ${Be|}_{Ns}$ as function of mass flow $\dot{m}$ for different temperatures *T_C_*. Experimental results for *D_h_* = 0.015 m, *L* = 0.1 m, $\dot{m}$ = 0.003 kg.s^−1^ and $\dot{m}$ = 0.004 kg.s^−1^.

As shown in Equations (4), (8) and (13), it appears that the greater the mass flow $\dot{m}$, the higher the fluid frictions and pressure drop. As a result, at a fixed mass flow, both entropy generation rate *N_S_* and irreversibility distribution ratio *ϕ* increase with the heat transfer (Figure 13). Effectively, at low temperatures (*T_C_* = 240 K), the heat transfer from the coolant at mean bulk temperature *T_mb_* = (*T_i_* + *T_o_*)/2 to the gas is higher than at high temperatures (*T_C_* = 260 K) and *Ns* presents highest variations. This observed for the two experimental temperatures *T_Cexp_* = 242 K and *T_Cexp_* = 248 K corresponding to the mass flow rates $\dot{m}$ = 0.003 kg.s^−1^ and $\dot{m}$ = 0.004 kg.s^−1^, respectively. Consequently, at a low temperature (*T_C_* = 240 K) and small cooling flow rate, the irreversibility distribution *ϕ* and Bejan number ${Be|}_{Ns}$ confirm that heat transfer dominates fluid frictions (Figure 14 and Figure 15). These phenomena are verified for both refrigerant flow rates $\dot{m}$ = 0.003 kg.s^−1^ and $\dot{m}$ = 0.004 kg.s^−1^.

At given temperatures *Tc* with 240 K < *T_C_* < 255 K, and for low mass flow rates, $\dot{m}$ < 0.10, the trend in the evolution of the Bejan number ${Be|}_{Ns}$ shows the dominance of heat transfer over viscous friction and ${Be|}_{Ns}$ → 1. Experimental tests with $\dot{m}$ = 0.003 kg.s^−1^ and $\dot{m}$ = 0.004 kg.s^−1^ are thus found in this working area of the refrigerating machine.

### 4.3. Effect of Cold Gas Temperature T_C_

**Figure 16:**
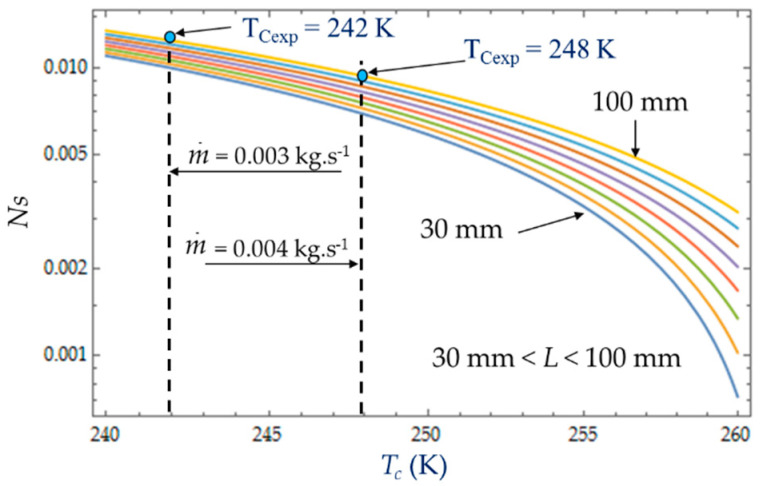
Variations in entropy generation rate *N_S_* as function of temperature *T_C_* for different lengths *L*. Experimental results for *D_h_* = 0.015 m, *L* = 0.1 m, $\dot{m}$ = 0.003 kg.s^−1^ and $\dot{m}$ = 0.004 kg.s^−1^.

**Figure 17:**
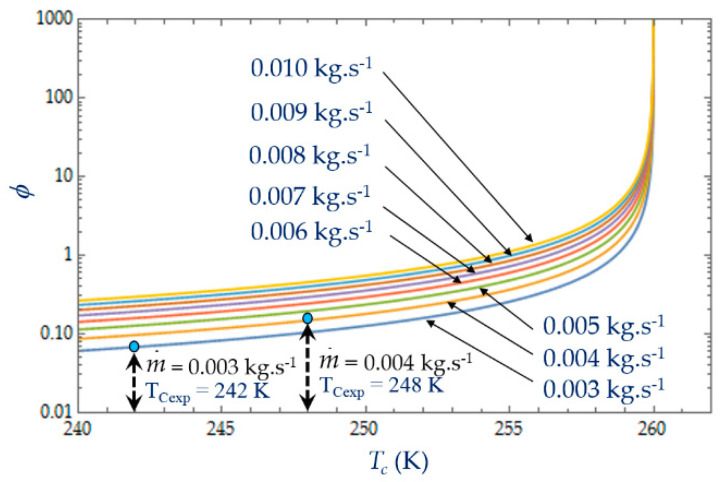
Variation in the irreversibility distribution ratio φ as function of temperature Tc for different mass flow $\dot{m}$. Experimental results for *D_h_* = 0.015 m, *L* = 0.1 m, $\dot{m}$ = 0.003 kg.s^−1^ and $\dot{m}$ = 0.004 kg.s^−1^.

The temperature *T_C_* of the cold gas inside the expansion volume is measured when the Stirling refrigerator runs at a steady rotational speed ω. For given inlet and outlet temperatures, *T_i_* = 300 K and *T_o_* = 260 K respectively, the heat transfer exchanged with the collant at mean bulk temperature decreases with the coolant temperature (Figure 16). This figure shows that, at the given cold gas temperature *Tc*, the entropy generation rate *N_S_* increases with the length *L* of the heat exchanger due to an increased pressure drop caused by viscous friction along the heat exchanger confirming the dominance of pressure drop [29,42]. As the amplitudes of gas temperature increase, both heat transfer exchanged with the coolant fluid at mean bulk temperature and irreversibility distribution ratio *φ* increase (Figure 17). At low temperatures (e.g., 240 K), the viscosity and the Prandlt number of the liquid increase sharply, with a factor around 7 between 243 K and 263 K, and the friction losses become high even at low flow rates. For the limit temperature *Tc* → 260 K, at fixed mass flow $\dot{m}$, the heat transfer would require an infinite quantity or a perfect heat exchanger efficiency and that is why the irreversibility distribution ratio *φ* rises sharply as the temperature approaches the limit *Tc* → 260 K and would tend to infinity for *Tc* = 260 K.

## 5. Conclusions

We have developed an analysis of the entropy generation rate inside a cold heat exchanger of a Stirling refrigerator by examining the behavior of three criteria: the adimensionless entropy ratio *N_S_*, the irreversibility distribution ratio *φ* and the Bejan number ${Be|}_{Ns}$ based on the adimensionless entropy ratio *N_S,_*_∆*T*_ and *N_S,_*_∆*P*_ corresponding to the heat transfer and fluid friction irreversibilities, respectively.

The cold heat exchanger is a double-pipe type heat exchanger made up of two concentric circular tubes. The incompressible coolant liquid flows continuously, with a mass flow rate, through the annular passage, under the temperature and pressure gradients, respectively. The other fluid is the working gas (Nitrogen) of the Stirling refrigerator and it flows alternatively (corresponding to the rotational frequency of the machine) at constant temperature T_C_ in the inner tube corresponding to the cylinder of the machine.

The cold heat exchanger transfers heat from a gas to a coolant liquid in the temperature range (240–300 K) and the efficiency of this operation is a result of the competition between heat transfer and fluid flow irreversibilities. We have carried out experiments with two mass flow rates $\dot{m}$ = 0.003 kg.s^−1^ and $\dot{m}$ = 0.004 kg.s^−1^ and showed the dominance of heat transfer over viscous friction in the cold heat exchanger, which presents a hydraulic diameter *D_h_* = 0.015 m and a length *L* = 0.10 m.

The aim of the analysis was to understand the impact of hydraulic diameter *D_h_*, length *L*, coolant mass flow $\dot{m}$ and cold gas temperature *T_C_* on these three criteria. It could be shown that, to ensure a minimal entropy production ratio *N_S_*, it is necessary to minimize the fluid friction irreversibilities (*N_S,_*_∆*P*_). Experimental results have shown the trade-off between fluid flow irreversibilities and heat transfer. We showed that large characteristic length and small hydraulic diameter generate large entropy production especially at low mean temperatures because of the high value of the coolant liquid (mix water/ethylene–glycol) viscosity increasing the fluid friction.

Our analysis needs to be further improved on these different points:

- Develop a second law analysis based on a heat exchanger submitted to periodic flows;
- Investigate other cold exchanger geometries with a larger hydraulic diameter and length and different mass flow rates;
- Perform exergetic and thermoeconomic analysis in order to define new indicators.

## Figures and Tables

**Table 1:** Mechanical characteristics of the Stirling refrigerator.

Characteristics	Values
Cooling capacity at 230 K/273 K	280 W/550 W
Cold end temperature	190 K < T_C_ < 273 K
Hot sink temperature	T_H_ = 300 K
Working gas	Nitrogen
Mean Pressure	15 bar < P < 18 bar
Stirling refrigerator overall dimensions Width × Height × Depth	19 × 46 × 18 cm
Power piston diameter	D = 60 mm
Power piston stroke	C = 40 mm
Compression swept volume	V_swc_ = 120 cm^3^
Regenerator	Stainless steel wire mesh
Rotational speed	35 rad.s^−1^ < ω < 80 rad.s^−1^

**Table 2:** Physical properties of of the coolant liquid (55/45 by mass ethylene glycol/water mixture) in the temperature range 240 K < *T* < 300 K [39].

Temperature		Thermal Conductivity	Density	Specific Heat Capacity	Dynamic Viscosity	Prandtl Number
T (°C)	T (K)	*λ* (W.m^−1^.K^−1^)	*ρ* (kg.m^−3^)	*c_p_* (J.kg^−1^.K^−1^)	*µ* (mPa.s)	*Pr*
−30	243.15	0.3300	1057.40	3220.2	500	4879.1
−25	248.15	0.3360	1057.40	3242.5	250	2412.6
−20	253.15	0.3377	1056.80	3264.8	130	1256.8
−15	258.15	0.3406	1056.00	3287.1	95	916.8
−10	263.15	0.3406	1055.00	3309.4	70	680.1
−5	268.15	0.3406	1053.00	3331.7	55	538.0
0	273.15	0.3440	1050.90	3354.0	40	390.0
5	278.15	0.3480	1048.25	3376.3	30	291.1
10	283.15	0.3507	1045.60	3398.6	20	193.8
15	288.15	0.3536	1042.65	3420.9		
20	293.15		1039.70	3443.2		
30	303.15		1033.80	3487.8		
